# LncRNA LYPLAL1-AS1 rejuvenates human adipose-derived mesenchymal stem cell senescence via transcriptional *MIRLET7B* inactivation

**DOI:** 10.1186/s13578-022-00782-x

**Published:** 2022-04-21

**Authors:** Yanlei Yang, Suying Liu, Chengmei He, Taibiao Lv, Liuting Zeng, Fengchun Zhang, Hua Chen, Robert Chunhua Zhao

**Affiliations:** 1 Department of Rheumatology and Clinical Immunology, Peking Union Medical College Hospital, Clinical Immunology Center, Chinese Academy of Medical Sciences and Peking Union Medical College, The Ministry of Education Key Laboratory, Beijing, China; 2 Institute of Basic Medical Sciences Chinese Academy of Medical Sciences, School of Basic Medicine Peking Union Medical College, Peking Union Medical College Hospital, Center of Excellence in Tissue Engineering Chinese Academy of Medical Sciences, Beijing Key Laboratory (No. BZO381), Beijing, China; 3 School of Life Sciences, Shanghai University, Shanghai, China

**Keywords:** hADSCs, Senescence, LYPLAL1-AS1, *MIRLET7B*, miR-let-7b

## Abstract

**Background:**

Mesenchymal stem cell (MSC) senescence is a phenotype of aging. Long noncoding RNAs (lncRNAs) are emerging as potential key regulators of senescence. However, the role of lncRNAs in MSC senescence remains largely unknown.

**Results:**

We performed transcriptome analysis in senescent human adipose-derived MSCs (hADSCs) and identified that the lncRNA LYPLAL1 antisense RNA1 (LYPLAL1-AS1) was significantly downregulated in senescent hADSCs. LYPLAL1-AS1 expression in peripheral blood was lower in middle-aged healthy donors than in young adult donors, and correlated negatively with age. Knockdown of LYPLAL1-AS1 accelerated hADSC senescence, while LYPLAL1-AS1 overexpression attenuated it. Chromatin isolation by RNA purification (ChIRP) sequencing indicated that LYPLAL1-AS1 bound to the *MIRLET7B* promoter region and suppressed its transcription activity, as demonstrated by dual-luciferase assay. miR-let-7b, the transcript of *MIRLET7B,* was upregulated during hADSC senescence and was regulated by LYPLAL1-AS1. Furthermore, miR-let-7b mimics promoted hADSC senescence, while the inhibitors repressed it. Finally, LYPLAL1-AS1 overexpression reversed miR-let-7b-induced hADSC senescence.

**Conclusions:**

Our data demonstrate that LYPLAL1-AS1 rejuvenates hADSCs through the transcriptional inhibition of *MIRLET7B*. Our work provides new insights into the mechanism of MSC senescence and indicates lncRNA LYPLAL1-AS1 and miR-let-7b as potential therapeutic targets in aging.

**Supplementary Information:**

The online version contains supplementary material available at 10.1186/s13578-022-00782-x.

## Introduction

Aging and aging-related chronic diseases such as cardiovascular and cerebrovascular diseases, central nervous diseases, and degenerative osteoarthritis are increasingly burdensome health issues. Stem cell exhaustion, impaired proliferation, and regenerative capacity are important causes of physiological and pathological aging [1, 2]. Mesenchymal stem cells (MSCs) originate in the mesoderm and are isolated from diverse tissues including adipose [3], bone marrow [4], the umbilical cord [5], and peripheral blood [6]. MSC populations and pools also decline with age, contributing to human aging and age-related diseases [7].

Given their self-renewal properties, multilineage differentiation potential, and extensive immunomodulatory effects, MSCs are promising tools for cell-based therapies for various diseases, including hematological diseases, autoimmune diseases, peripheral nerve injuries, cardiovascular diseases, and pulmonary infection [8–11], with numerous clinical trials currently underway [12], which demands extensive expansion of MSCs in vitro. However, like other cell types, MSCs undergo senescence in culture. Therefore, elucidating the molecular mechanisms of MSC senescence is essential for stem cell-based therapy in translational medicine.

Acquired senescence such as replicative senescence following extensive passaging including cell cycle arrest, impaired function, or loss of the regenerative phenotype limits the use of MSCs in aging-related disease [13–16]. Cellular senescence is a complex and potentially irreversible process driven by oxidative stress, DNA damage, telomere shortening, and oncogene activation [16]. Although MSCs have significant proliferative potential, they, as with other cells, present replicative senescence after multiple divisions, which is promoted by oxidative stressors such as hydrogen peroxide (H_2_O_2_) [17]. Senescent MSCs exhibit enlarged and granular morphology, deficient cell proliferation and differentiation capacity, produce the senescence-associated secretory phenotype (SASP), and have increased senescence-associated beta-galactosidase (SA-β-Gal) activity [16, 18]. The complex underlying mechanisms and regulatory networks of senescence remain to be fully elucidated. The P53–P21 and PRB–P16 pathways are two complementary senescence regulatory pathways that trigger and maintain cellular senescence [19].

Long noncoding RNAs (lncRNAs) are transcripts longer than 200 bp that lack protein-coding capacity. They are emerging as important and divergent regulators in biological processes through their interaction with chromatin modifiers, DNA, RNA, and RNA-binding proteins (RBPs) [20, 21]. Accumulating evidence shows that lncRNAs are altered during aging and senescence stimulation [22, 23]. A recent study using transcriptome profiling and loss-of-function screening showed that lncRNA-OIS1 is essential for oncogene-induced human fibroblast senescence by regulating CDKN1A and DPP4 [24]. Dong et al. revealed that lncRNA CYP7A1-1 is upregulated with age in human bone marrow-derived MSCs and that downregulating CYP7A1-1 rejuvenated aged bone marrow-derived MSCs through *SYNE1* [25]. However, the role of lncRNAs in MSC senescence remains largely unknown.

In the present study, we profiled the transcriptome in senescent human adipose-derived MSCs (hADSCs). We found that lncRNA LYPLAL1-AS1 was significantly downregulated in senescent hADSCs and was inversely associated with the age of healthy donors. We also demonstrate that LYPLAL1-AS1 was a negative regulator of hADSC senescence, which was potentially mediated by downregulating miR-let-7b levels by suppressing *MIRLET7B* promoter activity. Our findings provide new insight into the underlying mechanism of hADSC senescence and indicate potential therapeutic targets in anti-aging.

## Materials and methods

### Cell culture and differentiation

Human adipose-derived MSCs (hADSCs) were isolated from adipose tissues of healthy donors undergoing liposuction as described previously [26–28]. All experiments and procedures were approved by the Ethics Committee of Peking Union Medical College Hospital. hADSCs were maintained at a density of 1.7 × 10^5^ cells/ml in a 75 cm^2^ flask at 37 °C, 5% CO2 and were passaged with trypsin/EDTA on 90% confluence as we previously described in detail [29].

For adipogenic differentiation, hADSCs were incubated in adipogenic differentiation medium containing high glucose-DMEM (Gibco) supplemented with 10% FBS (Gibco), 1 µM dexamethasone (Sigma-Aldrich), 0.5 mM isobutylmethylxanthine (Sigma-Aldrich), and 1 mM ascorbic acid (Sigma-Aldrich) [30]. Adipogenic differentiation efficiency was determined by oil red O (Sigma-Aldrich) staining [31].

For osteogenic differentiation, hADSCs were incubated in H-DMEM supplemented with 10% FBS, 10 mM β-glycerophosphate (Sigma-Aldrich), 0.5 mM l-ascorbic acid (Sigma-Aldrich), and 0.01 mM dexamethasone [31]. ALP staining using ALP staining kit (Institute of Hematology and Blood Diseases Hospital, Chinese Academy of Medical Sciences, Tianjin, China) and Alizarin red staining [31] were used to measure osteogenic differentiation.

### Flow cytometry

hADSCs at passage 3 and passage 10 were harvested with 0.05% trypsin‐EDTA and washed twice using PBS (Servicebio) and then were incubated with phycoerythrin (PE)-conjugated antibodies against CD29, CD73, CD44, CD90, CD105, CD206, CD34, CD45, CD106, and HLA-DR, FITC-conjugated CD206 or isotype control antibodies (BD Biosciences) for 20 min at 4 °C in dark. Cells were washed twice and were analyzed on Accuri C6 flow cytometer (BD Biosciences) with CFlow Plus software (BD Biosciences)[29].

### Induction of hADSCs senescence

For replicative senescence, hADSCs were continuously cultured in normal medium in 75 cm^2^ flask and were passaged at 1:2 ratio normally following the hADSCs culture steps [29]. For H_2_O_2_-induced senescence, hADSCs at 70% confluence were treated with hydrogen peroxide (H_2_O_2_) (100 nM, 300 nM, and 500 nM) for 2 h [32], then cells were washed with PBS and were incubated in fresh media for 24–48 h. Cell senescence was evaluated by β-galactosidase assay using senescence β-galactosidase staining kit (YEASEN, Shanghai, China) according to manufacturer’s instructions. Briefly, cells were fixed for 15 min at room temperature in 4% paraformaldehyde, washed twice with PBS, then were incubated with SA-β-gal staining working solution overnight at 37 °C without CO_2_ in dark. The positive cells were stained blue, and images were acquired using an inverted microscope (Olympus, Japan).

### Clinical samples

Peripheral blood samples of healthy donors (n = 42) were provided by Peking Union Medical College Hospital. The study protocol was approved by the Ethics Committee of Peking Union Medical College Hospital and written informed consent was provided by all donors. The demographic data of donors was listed in Additional file 6: Table S1.

### Cell transfection

siRNA-NC, siRNA-LYPLAL1-AS1, mimics-NC, mimics-miR-let-7b, inhibitors-NC, inhibitors-miR-let-7b, at concentrations of 50 nM were transfected into hADSCs using lipofectamine 2000 (Invitrogen) according to manufacturer's protocol and as we previously described [29]. After 24–48 h of transfection, the hADSCs were collected for further analysis.

For overexpression, full-length LYPLAL1-AS1 was inserted into the LV5-EF1-a-EGFP-Puro lentivirus expression vector and packaged by GenePharma (Shanghai, China), and a lentiviral vector that expressed scrambled RNA was used as the negative control. hADSCs were infected with viral precipitates at a multiplicity of infection of 10, and stable cell lines were established by puromycin treatment as described previously [29].

### RNA extraction and quantitative real-time polymerase chain reaction (qRT-PCR)

Whole blood RNA was extracted by the MolPure Blood RNA Kit (YEASEN, Shanghai) according to manufacturer's instructions. Total RNA of cultured cells was extracted using TRIzol (Invitrogen) according to manufacturer’s instructions. cDNA was synthesized using M-MLV Reverse Transcriptase kit (Takara, Japan). qPCR was performed using SYBR-Green Master mix (YEASEN, Shanghai, China) on an QuantStudio Design & Analysis system (ABI, USA). Relative RNA levels were normalized to GAPDH expression using 2^−ΔΔCt^ method.

For miRNA detection, primers of miR-let-7b used for reverse transcription and qRT-PCR were designed by Sango Biotech (Shanghai, China), U6 was served as internal control. The primer sequences were listed in Additional file 6: Table S2.

### Transcriptome RNA sequencing

hADSCs from three donors were maintained in 6-well plates and were harvested at passage 3 and passage 10. RNA was extracted and library preparations and sequencing was processed on a Hiseq 4000 platform by Novogene (Beijing, China). Differential expression analysis was performed using the DESeq2 R package (1.20.0) with a false discover rate (FDR) cutoff of 0.05. The resulting P-values were adjusted using the Benjamini and Hochberg’s approach for controlling the false discovery rate. |log2FoldChange|≥ 2 and adjusted P-value < 0.05 were chosen as the cutoff criteria for differentially expressed genes. The RNA-Sequencing data is available at NCBI under SRA accession number PRJNA803433.

### Subcellular fractionation

Nuclear and cytosolic fractions was extracted using NE-PER Nuclear and Cytoplasmic Extraction Reagents (Thermo Fisher Scientific, USA) according to manufacturer’s instructions [33]. RNA was extracted and qRT-PCR was performed to assess the relative expression in nuclear and cytoplasmic fractions.

### *Fluorescent *in situ* hybridization (FISH) assay*

FISH was performed using an RNA FISH kit (RiboBio, China) as described previously [29]. Briefly, hADSCs cultured on coverslips were rinsed in PBS and fixed with 4% formaldehyde for 10 min. Cells were permeabilized in PBS containing 0.5% Triton X-100 at 4 °C for 5 min, washed three times, and prehybridized at 37 °C for 30 min. Then, anti-LYPLAL1-AS1, anti-U6, or anti-18S oligodeoxynucleotide probes (RiboBio, China) diluted in hybridization solution were incubated with cells in dark at 37 °C overnight. Cells were stained with DAPI and were imaged using a fluorescence microscope (Carl Zeiss, Germany).

### Chromatin isolation by RNA purification (ChIRP)-sequencing

hADSCs were subjected to ChIRP assay as described [29, 34]. Briefly, Antisense RNA probes were designed (Aksomics, China) to bind every 100 bp of LYPLAL1-AS1 and U1 (positive control) transcripts, with BiotinTEG biotin label conjugated to 3’end. hADSCs (2 × 10^7^) were fixed with 1% glutaraldehyde for RNA-chromatin cross-linking, and were snap-frozen in liquid nitrogen and stored at -80 °C. Samples were added to lysis buffer supplemented with protease inhibitor PMSF at room temperature, and cross-linked DNA-RNA complexes were sonicated until cell lysate was clear to break DNA into 100–500 bp fragments. Cell lysate was hybridized with biotinylated RNA probes. The extraction and subsequent analysis of RNA, DNA, and nucleic acid-binding proteins were performed. Sequencing analysis was performed by Aksomics (Shanghai, China). Sequence reads were generated from Illumina HiSeq 4000, image analysis and base calling were performed using Off-Line Basecaller software (OLB V1.8.0). After passing Solexa CHASTITY quality filter, the clean reads were aligned to Human genome (HG19) using BOWTIE software (V2.1.0); The mapped reads were used for peak detection by MACS V1.4.2 (Model-based Analysis of ChIRP-Seq) software. Statistically significant ChIRP-enriched regions (peaks) were identified by comparison of IP vs Input or comparison to a Poisson background model (Cut-off p-value = 10^–5^); The GO categories are derived from Gene Ontology (www.geneontology.org); Pathway analysis are based on the latest KEGG (Kyoto Encyclopedia of Genes and Genomes) database. The ChIRP-sequencing data is available at NCBI under SRA accession number PRJNA788657.

### Western blot

Protein was extracted using RIPA buffer with PMSF (1:100, Beyotime, China) and was quantified with BCA Protein Assay kit (Beyotime, China). Western blotting was performed in triplicates as previously described [33]. The following antibodies were used: P16 (1:1000, rabbit IgG; Proteintech, 10883-1-AP), P21 (1:1000, rabbit IgG; Proteintech, 10355-1-AP), P53 (1:1000, rabbit IgG; Proteintech, 10442-1-AP), LMNB1 (1:1000, mouse IgG; Proteintech, 66095-1-Ig). Horseradish peroxidase (HRP)-conjugated anti-rabbit-IgG, and HRP-conjugated anti-mouse-IgG (NeoBioscience).

### Dual-luciferase assay

*MIRLET7B* promoter sequence (2000 bp) was inserted into pGL4 basic luciferase reporter vectors (Promega, USA). Cells (5 × 10^5^) were transfected with 0.5 μg expression vector (pGL4-LET7B Promoter or pGL4-Basic), 0.5 μg pCDNA3.1 plasmid (LYPLAL1-AS1 or empty vector) or 5 μl LYPLAL1-AS1 siRNA. *MIRLET7B* promoter activity was normalized by co-transfection with 10 ng of Renilla luciferase reporter. After 48 h, luciferase activity was detected using Dual-Luciferase Reporter Assay System (Promega, USA). Firefly luciferase activity was normalized to Renilla luciferase activity to yield relative luciferase activity. The *MIRLET7B* promoter sequence was listed in Additional file 6: Table S3, all vectors are constructed by GenePharma (Shanghai, China).

### Statistical analysis

Data were expressed as mean ± standard deviation. Student’s t-test and One-way analysis of variance were used for comparisons between two groups and multiple groups, respectively. Statistically significant differences were defined as follows: *p < 0.05, ** *p* < 0.01, *** *p* < 0.001, and **** *p* < 0.0001. All statistical analysis was performed using GraphPad Prism7 software (GraphPad Prism, San Diego, CA).

## Results

### Characterization of hADSC senescence

**Fig. 1:**
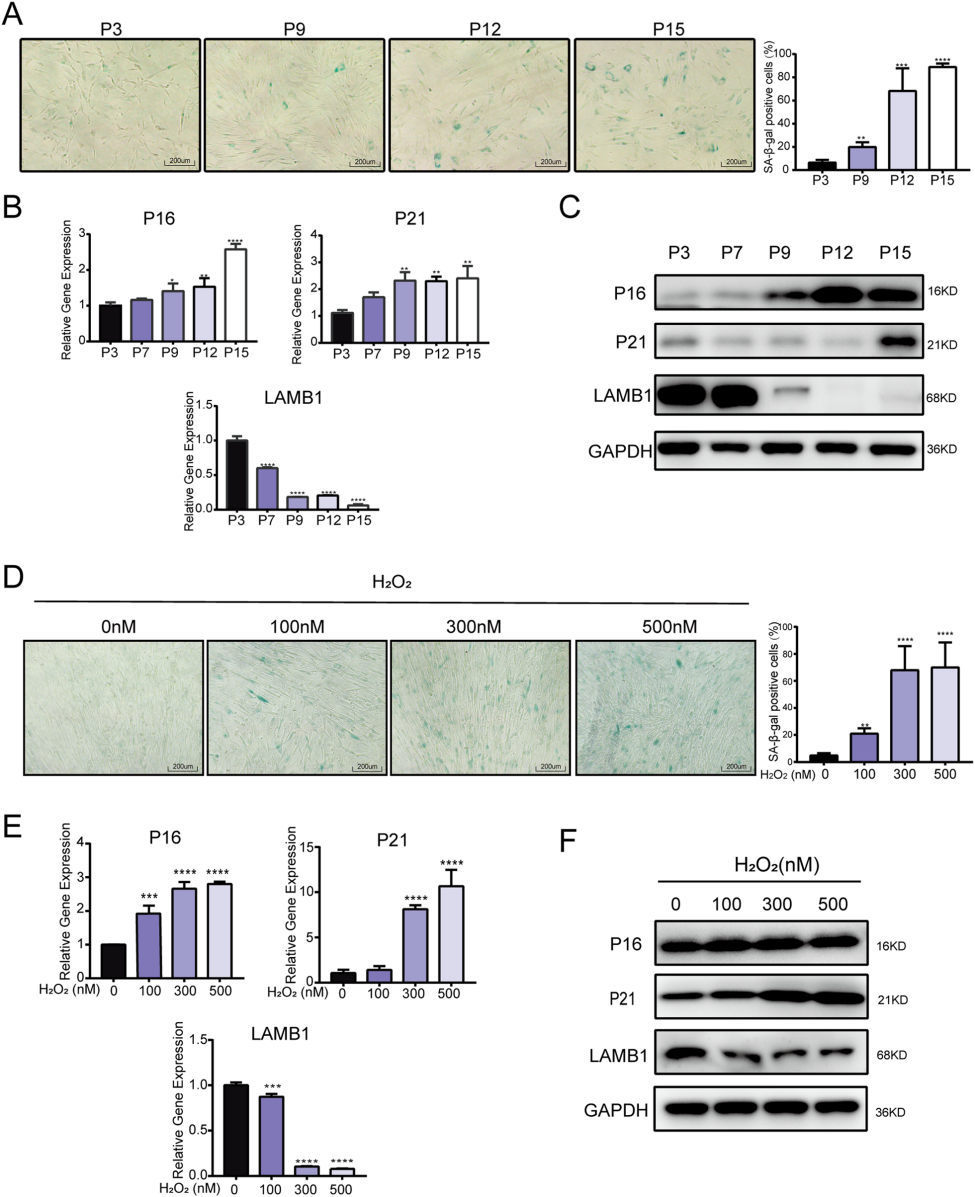
Characterization of hADSC senescence. **A** SA-β-Gal staining of hADSCs during replicative senescence at different passage(P): P3, P9, P12, and P15. **B** qRT-PCR of senescence markers *P16*, *P21*, and *LMNB1* in hADSCs during replicative senescence. **C** Western blot of P16, P21, and LMNB1 in hADSCs during replicative senescence. **D** SA-β-Gal staining of H_2_O_2_-treated hADSCs. **E** qRT-PCR of *P16*, *P21*, and *LMNB1* in H_2_O_2_-treated hADSCs. **F** Western blot of P16, P21, and LMNB1 in H_2_O_2_-treated hADSCs. qPCR data were normalized to *GAPDH*, n = 3. Data are shown as mean ± SD from three independent experiments. *p < 0.05, **p < 0.01, ***p < 0.001, ****p < 0.0001; scale bars: 200 µm

hADSCs are fibroblast-like plastic-adherent cells with the potential to differentiate into adipocytes and osteocytes. They express CD29, CD73, CD44, CD90, and CD105 but not CD106, CD206, CD34, CD45, and HLA-DR (Additional file 2: Fig. S1A–E). hADSCs senescence was induced in vitro via serial passaging in culture and H_2_O_2_-mediated oxidative stress [35, 36]. With increasing passage (P) numbers, the hADSCs gradually exhibited senescent morphology characterized by a flat and irregular shape, and increased SA-β-Gal staining (Fig. 1A). The hADSC phenotype remained stable from P3 to P10 (Additional file 2: Fig. S1A, S1B), while senescent hADSCs showed decreased differentiation potential from P3 to P10 (Additional file 2: Fig. S1C–S1E). qRT-PCR revealed that the senescence markers P16 and P21 were significantly increased, while the negative marker LMNB1 was significantly downregulated during hADSC serial passaging (Fig. 1B), which were confirmed by western blotting (Fig. 1C). Furthermore, SA-β-Gal-positive hADSCs increased as H_2_O_2_ concentrations increased (Fig. 1D). qRT-PCR confirmed that P16 and P21 were increased while LMNB1 was decreased in H_2_O_2_-treated hADSCs (Fig. 1E), which was confirmed by western blotting (Fig. 1F). These results indicate that serial passaging and H_2_O_2_-induced oxidative stress injury drove hADSCs to a senescent state.

### Downregulated LYPLAL1-AS1 in senescent hADSCs

**Fig. 2:**
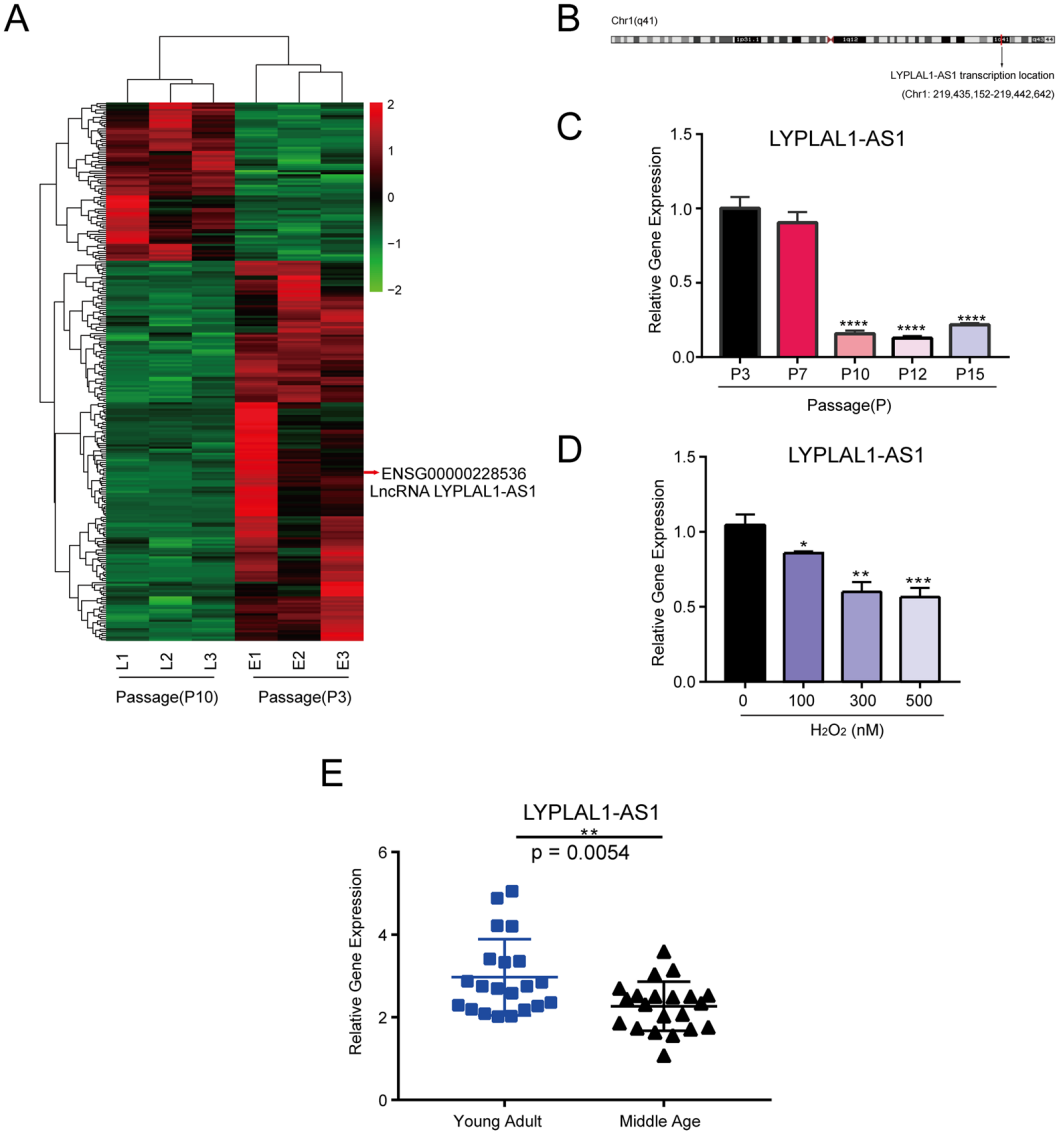
Downregulated LYPLAL1-AS1 in senescent hADSCs. **A** Heatmap of differentially expressed genes between P10 and P3 hADSCs from three donors. Red arrow indicates LYPLAL1-AS1. **B** Genomic location of LYPLAL1-AS1. **C** qRT-PCR of *LYPLAL1-AS1* in hADSCs during replicative senescence at different passages. **D** qRT-PCR of LYPLAL1-AS1 in hADSCs during replicative senescence at different passages. qRT-PCR of *LYPLAL1-AS1* in H_2_O_2_-treated hADSCs. **E** qRT-PCR of *LYPLAL1-AS1* in young adult (median age: 27 years, n = 21) and middle-aged healthy donors (median age: 53 years, n = 21). qPCR data were normalized to *GAPDH*, n = 3. Data are shown as mean ± SD from three independent experiments; *p < 0.05, **p < 0.01, ***p < 0.001, ****p < 0.0001

To systematically identify the transcriptome changes involved in hADSC senescence, we performed RNA sequencing analysis of P3 and P10 hADSCs derived from three donors. We identified 25,369 gene transcripts, including 75 upregulated genes and 180 downregulated genes in P10 hADSCs compared with P3 hADSCs (fold change ≥ 2, expression value ≥ 3, *p* < 0.05) (Fig. 2A). Among the differentially expressed genes, we noted that a lncRNA, LYPLAL1-AS1, which is transcribed in the opposite direction relative to *LYPLAL1* on chromosome 1, was notably downregulated in senescent hADSCs (Fig. 2A, B). qRT-PCR confirmed that LYPLAL1-AS1 was downregulated in hADSCs during serial passaging (Fig. 2C) and under H_2_O_2_ exposure (Fig. 2D). To validate the clinical relevance between LYPLAL1-AS1 and aging, we analyzed its expression in the peripheral blood of young adult (age: 24–28 years, median age: 27 years, n = 21) and middle-aged adult (age: 49–57 years, median age: 53 years, n = 21) healthy donors (Additional file 6: Table S1). qRT-PCR showed that LYPLAL1-AS1 expression was significantly higher in the young adults compared with the middle-aged adults (*p* = 0.0054) (Fig. 2E) and was inversely correlated with age (*r* = -0.3704, *p* = 0.0158) (Additional file 3: Fig. S2). Together, LYPLAL1-AS1 was downregulated in senescent hADSCs and older people, suggesting as a potential negative regulator of hADSC senescence.

### LYPLAL1-AS1 knockdown accelerates hADSC senescence

**Fig. 3:**
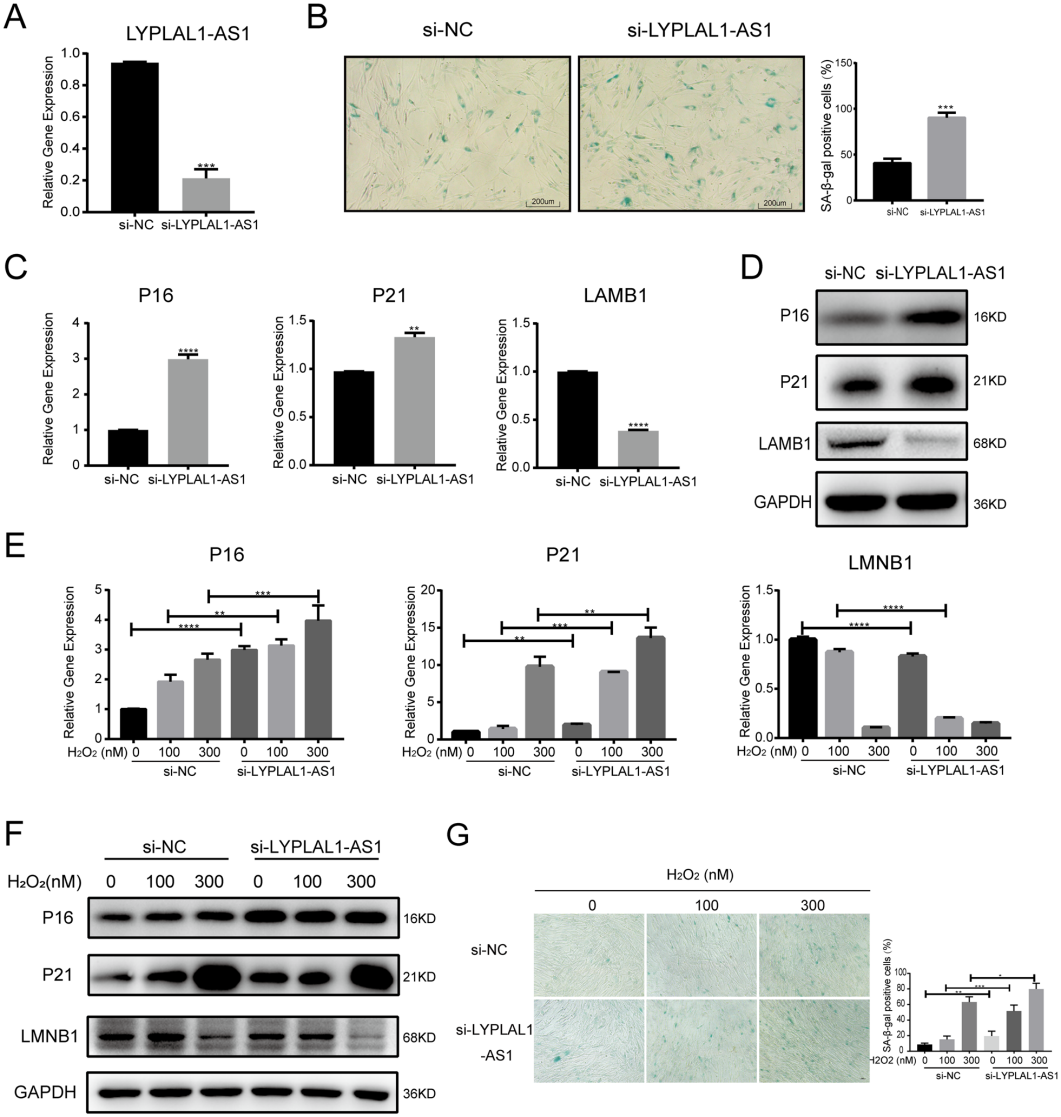
LYPLAL1-AS1 knockdown accelerates hADSC senescence. **A** qRT-PCR of siRNA-mediated knockdown of LYPLAL1-AS1. **B** SA-β-Gal staining of control and LYPLAL1-AS1-silenced P10 hADSCs. **C** qRT-PCR of P16, P21, and LMNB1 in control and LYPLAL1-AS1-silenced P10 hADSCs. **D** Western blot of P16, P21, and LMNB1 in control and LYPLAL1-AS1-silenced P10 hADSCs. **E** qRT-PCR of P16, P21, and LMNB1 in control and LYPLAL1-AS1-silenced H_2_O_2_-treated hADSCs. **F** Western blot of P16, P21, and LMNB1 in control and LYPLAL1-AS1-silenced H_2_O_2_-treated hADSCs. **G** SA-β-Gal staining in control and LYPLAL1-AS1-silenced H_2_O_2_-treated hADSCs. qPCR data were normalized to *GAPDH*, n = 3. Data are shown as mean ± SD from three independent experiments. *p < 0.05, **p < 0.01, ***p < 0.001, ****p < 0.0001; scale bars: 200 µm

To evaluate the role of LYPLAL1-AS1 in hADSC senescence, we first silenced LYPLAL1-AS1 in P10 hADSCs using specific small interfering RNAs (siRNAs) (Fig. 3A). SA-β-Gal-positive cells were significantly increased in the LYPLAL1-AS1-silenced hADSCs than in control hADSCs (Fig. 3B). qRT-PCR and western blotting indicated that P16 and P21 were upregulated, while LMNB1 was downregulated in LYPLAL1-AS1-silenced hADSCs (Fig. 3C, D). Furthermore, we silenced LYPLAL1-AS1 in H_2_O_2_-treated hADSCs. Consistent with the earlier results, qRT-PCR and western blotting revealed that LYPLAL1-AS1 knockdown enhanced the senescence effect of H_2_O_2_ on hADSCs, as indicated by increased P16 and P21 levels and decreased LMNB1 levels in the LYPLAL1-AS1 knockdown group (Fig. 3E, F), and by the decreased SA-β-Gal-positive cells (Fig. 3G). These data demonstrate that LYPLAL1-AS1 downregulation accelerates hADSC senescence.

### LYPLAL1-AS1 overexpression attenuates hADSC senescence

**Fig. 4:**
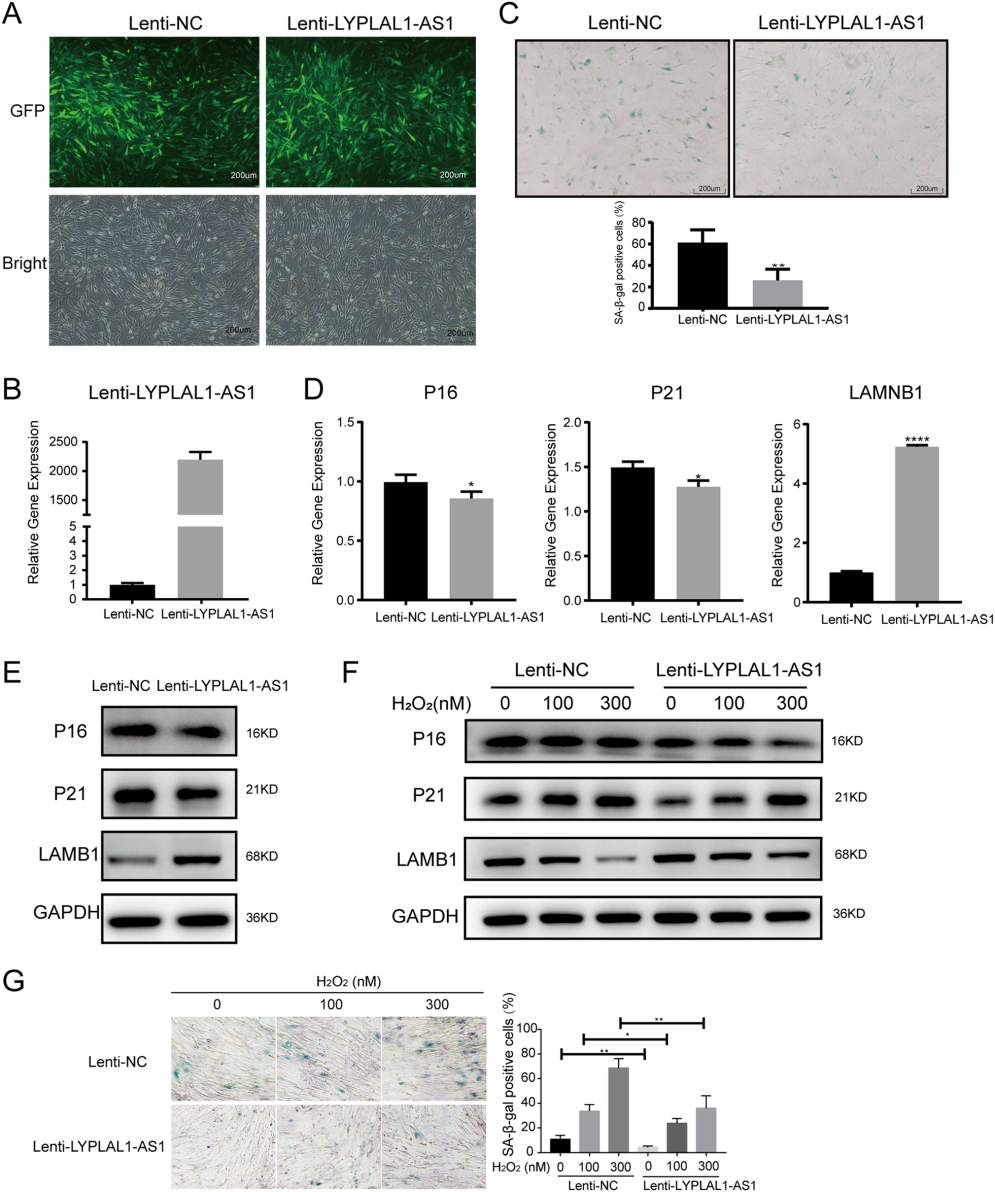
LYPLAL1-AS1 overexpression attenuates hADSC senescence. **A** Representative images of hADSCs transfected with lentivirus expressing GFP and control (Lenti-NC) or LYPLAL1-AS1 (Lenti-LYPLAL1-AS1). **B** qRT-PCR of overexpression efficiency of LYPLAL1-AS1 in hADSCs transfected with control or LYPLAL1-AS1 overexpression lentivirus. **C** SA-β-Gal staining in control and LYPLAL1-AS1 overexpression P10 hADSCs. **D **qRT-PCR of *P16*, *P21*, and *LMNB1* in control and LYPLAL1-AS1 overexpression P10 hADSCs. **E** Western blot of P16, P21, and LMNB1 in control and LYPLAL1-AS1 overexpression P10 hADSCs. **F** Western blot of P16, P21, and LMNB1 in control and LYPLAL1-AS1 overexpression H_2_O_2_-treated hADSCs. **G** SA-β-Gal staining in control and LYPLAL1-AS1 overexpression H_2_O_2_-treated hADSCs. qPCR data were normalized to *GAPDH*, n = 3. Data are shown as mean ± SD from three independent experiments; *p < 0.05, **p < 0.01, ****p < 0.0001; scale bars: 200 µm

To confirm the role of LYPLAL1-AS1 in hADSC senescence, we overexpressed LYPLAL1-AS1 in hADSCs using lentivirus. The lentiviral transfection efficiency was > 90% as determined by green fluorescent protein (GFP) expression (Fig. 4A), and LYPLAL1-AS1 was significantly overexpressed (Fig. 4B). LYPLAL1-AS1 overexpression decreased SA-β-Gal staining in senescent hADSCs (Fig. 4C) and impaired P16 and P21 expression while improving LMNB1 expression as detected by qRT-PCR (Fig. 4D) and western blotting (Fig. 4E). Western blotting also showed that LYPLAL1-AS1 overexpression inhibited the senescence effect of H_2_O_2_ on hADSCs, as P16 and P21 were decreased and LMNB1 was increased (Fig. 4F), and SA-β-Gal-positive cells were decreased (Fig. 4G). Collectively, these data indicate that LYPLAL1-AS1 overexpression inhibits hADSC senescence.

### LYPLAL1-AS1-regulated genes identified by ChIRP-seq

**Fig. 5:**
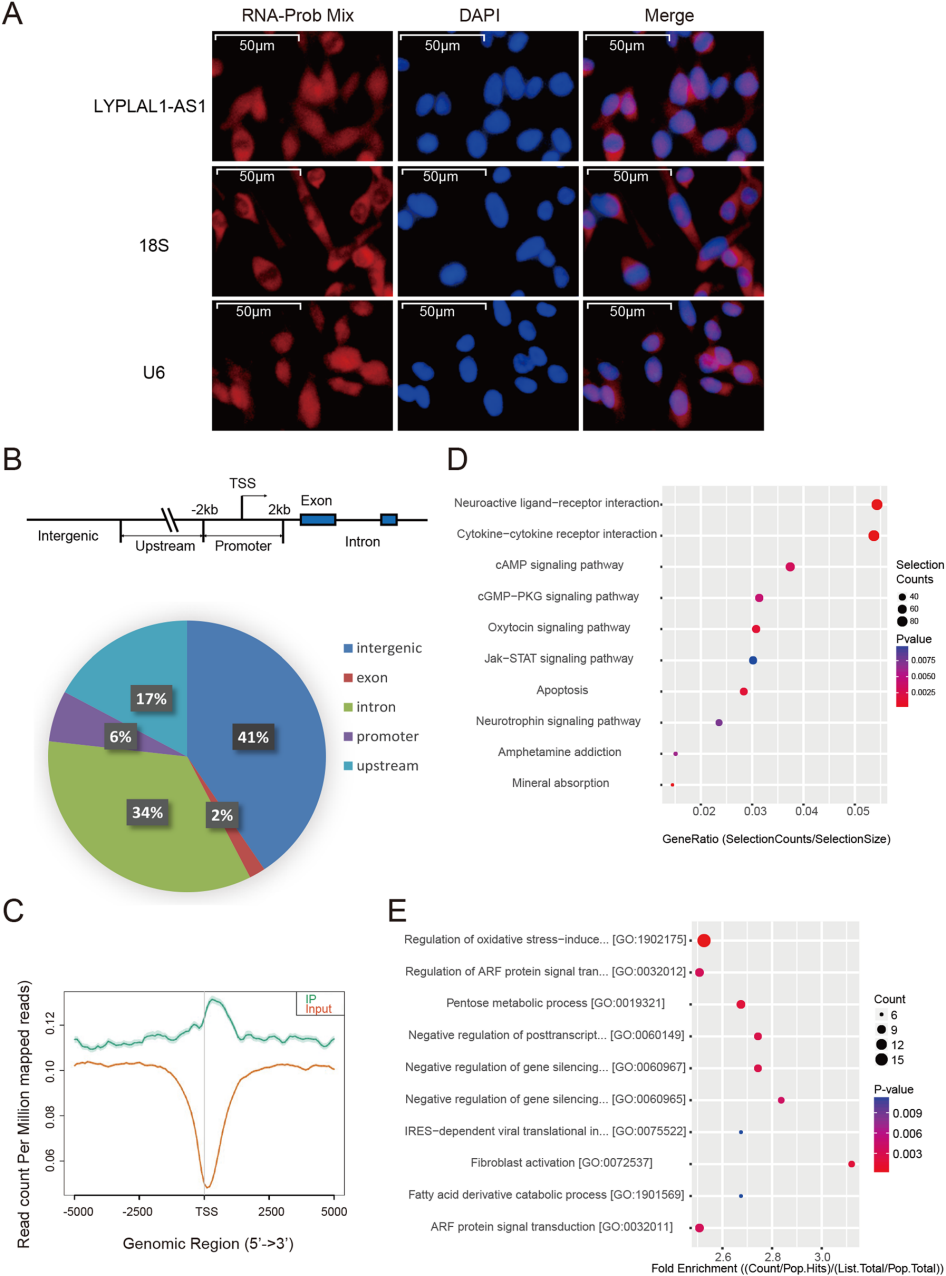
LYPLAL1-AS1-regulated genes identified by ChIRP-seq. **A** RNA fluorescence in situ hybridization (FISH) assay for LYPLAL1-AS1 in hADSCs. 18S and U6 were the cytoplasmic and nuclear RNA controls, respectively. Scale bars: 50 µm. **B** Diagram of the distribution of LYPLAL1-AS1 binding loci. **C** Distribution of peaks within the transcription start site (TSS) ± 5 kb of the LYPLAL1-AS1 binding-related elements. **D** KEGG pathway analysis of LYPLAL1-AS1-related genes. **E** GO biological process enrichment analysis of LYPLAL1-AS1-related genes

LYPLAL1-AS1 was distributed in both the nucleus and cytoplasm (Fig. 5A and Additional file 4: Fig. S3). To explore the downstream genes regulated by LYPLAL1-AS1, we conducted chromatin isolation by RNA purification sequencing (ChIRP-seq) analysis of LYPLAL1-AS1 in hADSCs to capture its direct binding DNA targets, which showed that LYPLAL1-AS1 bound at the intron, exon, promoter, upstream regions, and intergenic sites (Fig. 5B, C). We performed Gene Ontology (GO) and Kyoto Encyclopedia of Genes and Genomes (KEGG) pathway enrichment analyses on all binding genes. KEGG pathway enrichment analysis suggested that LYPLAL1-AS1 potentially regulated the senescence-related pathways, including the JAK–STAT signaling pathway and the apoptosis pathway (Fig. 5D). GO analysis revealed that the target genes of LYPLAL1-AS1 participated in diverse biological processes, including regulation of oxidative stress-induced genes and regulation of ARF protein signal transduction (Fig. 5E).

### LYPLAL1-AS1 negatively regulates MIRLET7B

**Fig. 6:**
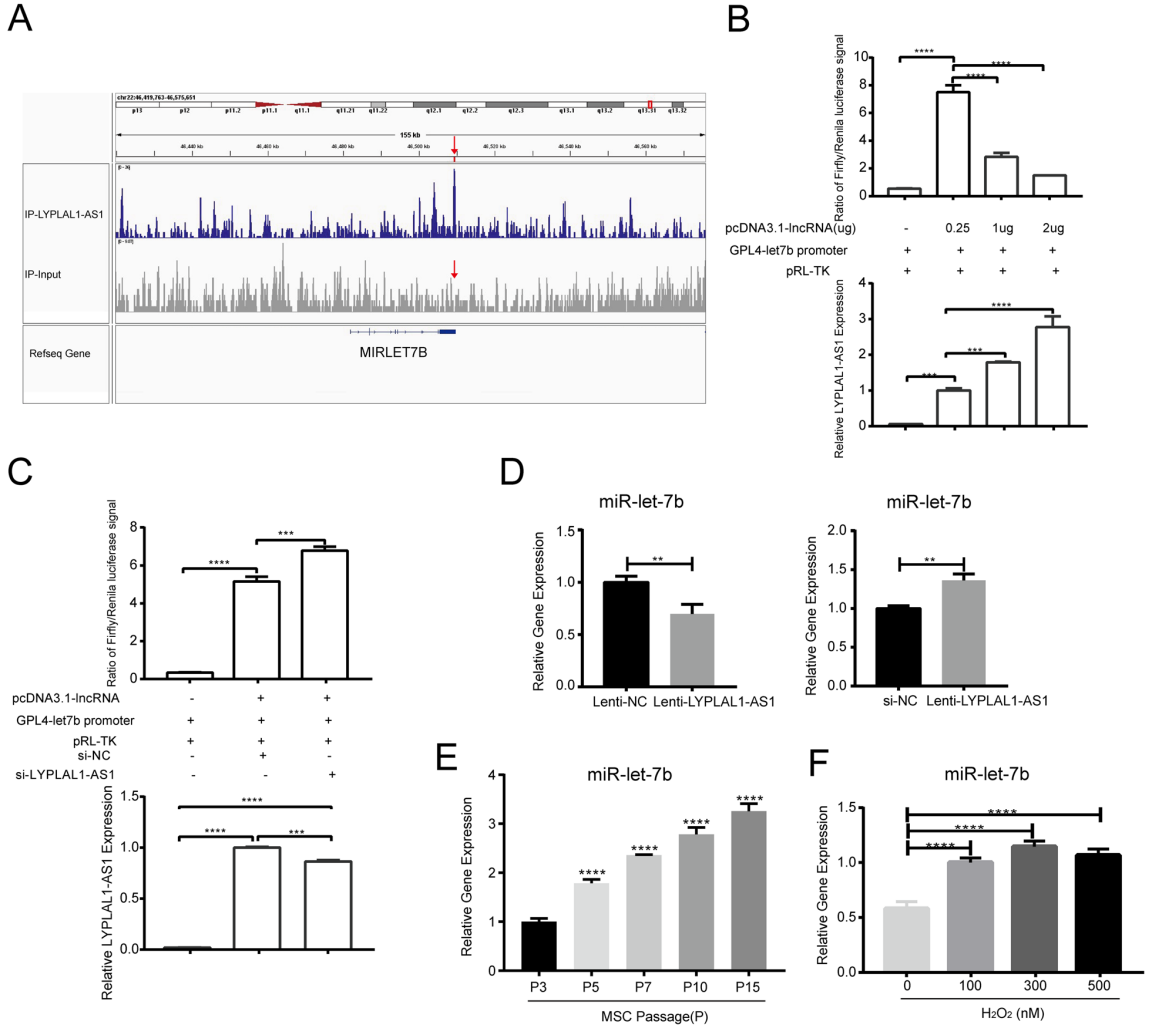
LYPLAL1-AS1 negatively regulates MIRLET7B. **A** Genome visualization of the LYPLAL1-AS1 binding site within the *MIRLET7B* promoter region detected by ChIRP-seq. **B** The relative firefly to *Renilla* luciferase activity (upper panel, n = 3) and LYPLAL1-AS1 expression (lower panel) of 293 T cells co-transfected with *MIRLET7B* promoter reporter vector (GPL4-let7b promoter), LYPLAL1-AS1 expression vector (pcDNA3.1-LYPLAL1-AS1), and *Renilla* luciferase vector (pRL-TK). **C** The relative firefly to *Renilla* luciferase activity (upper panel, n = 3) and LYPLAL1-AS1 expression (lower panel) of 293 T cells co-transfected with *MIRLET7B* promoter reporter vector (GPL4-let7b promoter), LYPLAL1-AS1 expression vector (pcDNA3.1-LYPLAL1-AS1), *Renilla* luciferase vector (pRL-TK), and LYPLAL1-AS1 siRNAs or control siRNAs. **D** qRT-PCR of miR-let-7b in LYPLAL1-AS1-silenced or -overexpressed hADSCs. **E** qRT-PCR of miR-let-7b in hADSCs during replicative senescence. **F** qRT-PCR of miR-let-7b in H_2_O_2_-treated hADSCs. qPCR data were normalized to *GAPDH*, n = 3. Data are shown as mean ± SD from three independent experiments; **p < 0.01, ***p < 0.001, ****p < 0.0001

ChIRP-seq showed that LYPLAL1-AS1 bound on the *MIRLET7B* promoter in a 1.2-kb window on chr22, which was presented by Integrative Genomics Viewer (IGV) software (Fig. 6A), suggesting that LYPLAL1-AS1 may regulate *MIRLET7B* transcription. *MIRLET7B* is the host gene of miR-let-7b-5p (hereafter referred to as miR-let-7b) that is augmented during oxygen exposure, causing oxidative stress and senescence in the choroid and retinal pigment epithelium through the p53–let-7b–IGF-1R axis [37], but the role of miR-let-7b in cell senescence remains unclear. Accordingly, we aimed to reveal whether *MIRLET7B* and its mature miRNA (miR-let-7b) are involved in LYPLAL1-AS1 repression hADSC senescence. To explore the transcriptional regulatory effect of LYPLAL1-AS1 on *MIRLET7B*, we transfected 293 T cells with *MIRLET7B* promoter–luciferase reporter vectors and LYPLAL1-AS1 expression vectors or empty vectors. The luciferase activity of the *MIRLET7B* promoter decreased as LYPLAL1-AS1 expression increased (Fig. 6B). Silencing LYPLAL1-AS1 induced enhanced *MIRLET7B* promoter–reporter activity (Fig. 6C), confirming that LYPLAL1-AS1 was a negative regulator of *MIRLET7B* promoter. Next, we evaluated whether LYPLAL1-AS1 regulated miR-let-7b expression levels, and profiled miR-let-7b expression during hADSC senescence. qRT-PCR showed that LYPLAL1-AS1 downregulation increased miR-let-7b expression, while LYPLAL1-AS1 upregulation decreased it (Fig. 6D). Accordingly, miR-let-7b expression was markedly increased in hADSCs with serial passaging (Fig. 6E) or H_2_O_2_ treatment (Fig. 6F), suggesting that miR-let-7b is a positive regulator of hADSC senescence. Collectively, these data reveal that LYPLAL1-AS1 directly bound to the *MIRLET7B* promoter and potentially downregulates miR-let-7b levels by suppressing *MIRLET7B* promoter activity.

### miR-let-7b promotes hADSC senescence, which is reversed by LYPLAL1-AS1

**Fig. 7:**
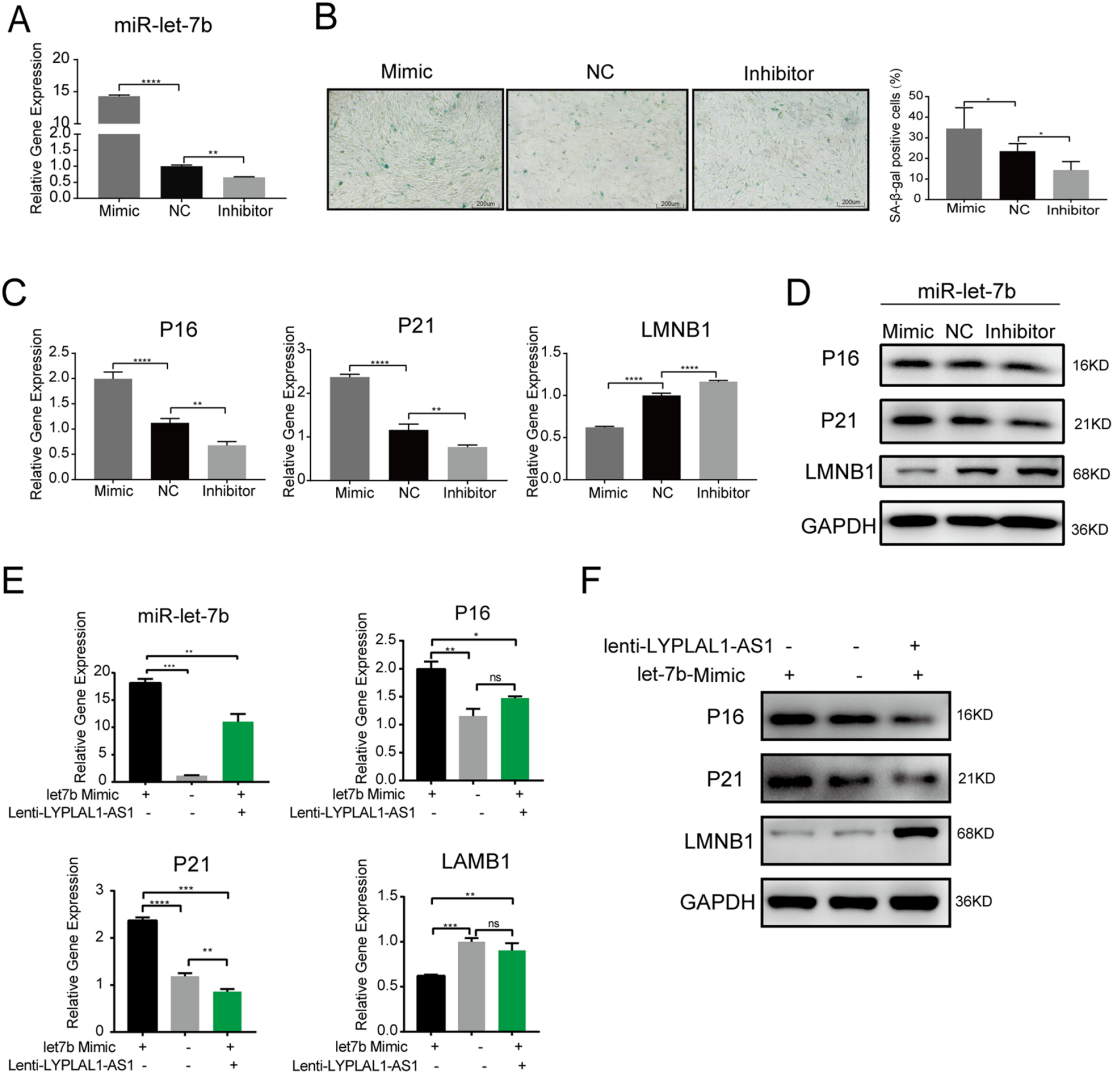
miR-let-7b promotes hADSC senescence, which is reversed by LYPLAL1-AS1. **A** qRT-PCR of miR-let-7b in hADSCs transfected with miR-let-7b mimic, NC, or miR-let-7b inhibitor. **B** SA-β-Gal staining in hADSCs transfected with miR-let-7b mimic, NC, or miR-let-7b inhibitor. **C **qRT-PCR of *P16*, *P21*, and *LMNB1* in hADSCs transfected with miR-let-7b mimic, NC, or miR-let-7b inhibitor. **D** Western blot of P16, P21, and LMNB1 in hADSCs transfected with miR-let-7b mimic, NC, or miR-let-7b inhibitor. **E** qRT-PCR of miR-let-7b, P16, P21, and LMNB1 in hADSCs transfected with miR-let-7b mimic and (or) LYPLAL1-AS1 expression vector. **F** Western blot of P16, P21, and LMNB1 in hADSCs transfected with miR-let-7b mimic and (or) LYPLAL1-AS1 expression vectors. qPCR data were normalized to *GAPDH*, n = 3. Data are shown as mean ± SD from three independent experiments; *p < 0.05, **p < 0.01, ***p < 0.001, ****p < 0.0001; scale bars: 200 µm

We explored whether miR-let-7b regulated hADSC senescence by transfecting miR-let-7b mimics, negative controls (NC), or miR-let-7b inhibitor into hADSCs. The miR-let-7b mimics increased miR-let-7b expression while the inhibitors decreased it (Fig. 7A). The miR-let-7b mimics promoted SA-β-Gal expression, while the miR-let-7b inhibitors attenuated it (Fig. 7B). The miR-let-7b mimics promoted hADSC senescence as shown by the upregulated P16 and P21 and downregulated LMNB1, while its inhibitors inhibited hADSC senescence as shown by the downregulated P16 and P21 and upregulated LMNB1 (Fig. 7C, D). The findings suggest that miR-let-7b is a positive regulator of hADSC senescence. To examine whether LYPLAL1-AS1 regulated miR-let-7b-induced hADSC senescence, we overexpressed LYPLAL1-AS1 or control in hADSCs then transfected them with miR-let-7b mimics. LYPLAL1-AS1 overexpression significantly inhibited the upregulated P16 and P21 levels and increased the downregulated LMNB1 level induced by the miR-let-7b mimics (Fig. 7E, F), indicating that LYPLAL1-AS1 reversed miR-let-7b-induced hADSC senescence. Collectively, our data indicate that LYPLAL1-AS1 is a negative regulator of hADSC senescence, potentially through transcriptional modulation of *MIRLET7B* expression.

## Discussion

Elucidating the regulation, intervention, and rejuvenation of MSC senescence is important for MSC-based therapy for anti-aging. Repeated subculture and H_2_O_2_ exposure induce MSC senescence, which are suitable models of MSC senescence in vitro [32, 38]. In the present study, we demonstrate that LYPLAL1-AS1 is a negative regulator of hADSC senescence induced by repeated subculture and H_2_O_2_ exposure, which was inversely correlated with aging. We also reveal that LYPLAL1-AS1 rejuvenates hADSC senescence through transcriptional inhibition of the *MIRLET7B* promoter.

LncRNAs have versatile regulation functions at multiple levels. LncRNA ANCR suppresses epidermal and definitive endoderm differentiation [33] and modulates osteogenic and adipogenic differentiation and tumor progression. LncRNA MEG3 promotes MSC osteogenic differentiation at transcriptional level by acting as a decoy to dissociate SOX2 binding at the *BMP4* promoter to activate BMP4 [39], and acts as a competing endogenous RNA (ceRNA) to regulate osteogenic gene expression at post-transcriptional level [40]. LncRNA-p21 is distributed in both the cytoplasm and nuclear, coordinates with RCK RNA helicase to suppress target mRNA translation in the cytoplasm [41], and methylates and maintains H3K9me3 at the promoters of pluripotent genes to repress their transcription by interacting with heterogeneous nuclear ribonucleoprotein (hnRNP) K protein [42]. We have reported that LYPLAL1-AS1 is a key promoter of hADSC adipogenic differentiation by directly targeting and modulating DSP protein stability in the cytoplasm [29]. Herein, we demonstrate that LYPLAL1-AS1 participates in regulating hADSCs senescence, which was mediated by the transcriptional inhibition of *MIRLET7B*. Therefore, LYPLAL1-AS1 regulates hADSCs at multiple levels, and other potential functions of LYPLAL1-AS1 on hADSCs remained to be explored. In addition, the function of specific lncRNAs is not restricted to a specific protein and they may influence multiple potential targets in a cell type-dependent way. For instance, lncRNA HOTAIR participates in regulating MSC function, which may cause senescence-associated DNA methylation to impact MSC proliferation and differentiation [43]. In senescent human fibroblasts, HOTAIR is upregulated and prevents premature senescence by causing rapid decay of targets Ataxin-1 and Snurportin-1 [44]. In cancer cell lines, HOTAIR depletion may be correlated with cell cycle arrest by decreasing S phase cells, inhibiting cell proliferation rate, and promoting apoptotic levels [45, 46]. Our study revealed one function of LYPLAL1-AS1 in preventing human adipose-derived-MSC (hADSC) senescence, and besides adipose tissue, LYPLAL1-AS1 also exhibits high expression in ovary, mammary and subcutaneous according to human RNA sequencing (RNA-Seq) expression data from UCSC (http://genome.ucsc.edu), therefore, extending our research to other cell types is a promising direction in the future.

MSC senescence is a complex process. Besides the innate molecular dynamic changes in SA-β-Gal staining and P16, P21, and LMNB1 expression examined in the present research, their functional abilities might also change, including the differentiation property which is intricately regulated by multiple factors, including transcriptional factors, growth factors, and epigenetic factors such as ncRNAs (miRNAs, lncRNAs) [47–49]. Our previous research indicate that LYPLAL1-AS1 is a key regulator of adipogenic differentiation, promoting the differentiation of normal hADSCs toward adipogenic cells by directly targeting and modulating DSP protein stability [29]. As shown in Additional file 5: Fig. S4A, overexpression of LYPLAL1-AS1 partially reversed the adipogenic differentiation of senescent hADSCs, as indicated by oil red O staining, suggesting that LYPLAL1-AS1 overexpression ameliorates hADSC senescence, resulting in increased cell differentiation potential. The differentiation potential of MSCs changed with senescence [50, 51]. Increasing evidence indicates that senescent MSCs exhibit elevated adipogenesis at the expense of osteogenesis which is consistent with the in vivo increased bone marrow adiposity in aging, leading to damaged bone formation ability in humans and animals [51–53]. Most studies indicate that osteogenic differentiation potential of MSCs deteriorates with age [38, 54, 55], and the adipogenic differentiation potential of MSCs is disputable. Some studies indicate that the adipogenesis potential of MSCs tends to decline with senescence [56, 57]. In a well-controlled study using rat model, MSCs undergoing long-term passaging exhibit impaired in vitro differentiation potential including complete loss of osteogenic potential and attenuated adipogenic potential [58]. Other studies indicate the increasing adipogenic potential of senescent MSCs [59–61]. The discrepancy about whether senescent MSCs harbor increased or decreased adipogenic potential may be due to the different in vitro culture conditions and complex in vivo microenvironment including cell-intrinsic cytokines and hormones. Therefore, cellular senescence might not adequately explain the change in MSC differentiation, and the cell-intrinsic mechanism that regulates the age-related change in MSC differentiation deserved comprehensive studies in the future. Besides, deficiency in cell proliferation is also a characteristic of senescent MSCs. We also found knockdown and overexpression of LYPLAL1-AS1 had little effect on the proliferation of young (P3) hADSCs (Additional file 5: Fig. S4B, C), while its overexpression significantly reversed the proliferation of senescent hADSCs (Additional file 5: Fig. S4C, D), suggesting that LYPLAL1-AS1 overexpression ameliorates cellular senescence by rejuvenating senescent hADSCs, resulting in increased cell proliferation. Future studies should explore the underlying cellular and molecular defects that may explain the altered cell proliferation in senescent hADSCs.

A common lncRNA–miRNA interaction mechanism is lncRNAs acting as sponges to compete with mRNAs for miRNA binding, also known as the ceRNA mechanism [62–64]. However, TargetScan predicted no binding sequence between LYPLAL1-AS1 and miR-let-7b (data not shown). Combined with the ChIRP-seq analysis showing direct binding between LYPLAL1-AS1 and the *MIRLET7B* promoter, we propose that LYPLAL1-AS1 inhibits *MIRLET7B* promoter activity, thereby indirectly modulating miR-let-7b levels at the transcriptional level. LncRNA regulation of gene transcription has been extensively studied [65, 66]. LncRNAs may act as guides to recruit proteins to DNA through RNA–DNA and RNA–protein interactions [67–69]. Guide lncRNAs have both protein-binding functions and genome-interfacing functions. Here, we reveal that LYPLAL1-AS1 interfaced directly with the *MIRLET7B* promoter and regulate its activity negatively. Further studies are warranted to determine whether other proteins are involved in this process. Therefore, we suggest a new underlying mechanism of LYPLAL1-AS1 inhibition of hADSC senescence: it potentially inhibits the *MIRLET7B* promoter transcriptionally, thereby downregulating miR-let-7b levels.

Similar to protein-coding genes, miRNA host gene promoters contain CpG islands, TATA box sequences, initiation elements, and certain histone modifications that enable their control by transcription factors, enhancers, silencing elements, and chromatin modifications [70, 71]. miRNA processing from the host gene or miRNA precursor transcription may affect mature miRNA levels [72]. Therefore, transcriptional regulation of miRNAs is partially responsible for their specific spatial and temporal expression patterns [73]. For example, p53 promotes miR-34 and miR-107 family expression, which enhances tumor cell cycle arrest and apoptosis [70, 74]. PITX3 stimulates miR-133b transcription and contributes to the maturation and function of midbrain dopaminergic neurons [75]. Herein, we found that LYPLAL1-AS1 downregulated miR-let-7b levels by binding to the promoter region of its host gene *MIRLET7B* and suppressing transcriptional activity, thereby attenuating hADSC senescence. miR-let-7b is a let-7 family miRNA that is upregulated in the plasma of patients with age-related macular degeneration [76]. A recent study has indicated that miR-let-7b is augmented in choroid and retinal pigment epithelium exposed to oxygen and that increased p53–miR-let-7b activity promotes the inability of the choroid to revascularize in oxygen-induced retinopathy [37]. However, whether miR-let-7b plays a role in hADSC senescence remains unknown. Our data demonstrate that miR-let-7b accelerates hADSC senescence, which is regulated by LYPLAL1-AS1. Therefore, targeting miR-let-7b might be a promising approach for reducing hADSC senescence.

## Conclusions

We demonstrate that LYPLAL1-AS1 rejuvenates hADSCs through the transcriptional inhibition of *MIRLET7B*. Our work provides new insights into the mechanism of stem cell senescence. LYPLAL1-AS1 and miR-let-7b might be potential therapeutic targets in aging-related diseases.

## Supplementary Information

**Additional file 1**. Methods and figure legends

**Additional file 2: Figure S1**. Morphological, functional, and phenotype characteristics of hADSCs

**Additional file 3: Figure S2**. Correlation analysis of LYPLAL1-AS1 expression level and age in healthy donors (n=42). data were analyzed using R software version 3.5.3

**Additional file 4: Figure S3**. Subcellular fractionation of LYPLAL1-AS1 in hADSCs followed by qRT-PCR. GAPDH and U6 mRNA served as cytoplasmic and nuclear control, respectively.

**Additional file 5: Figure S4**. LYPLAL1-AS1 overexpression ameliorates cellular senescence, resulting in increased cell differentiation potential and increased cell proliferation. A. Oil red O staining of adipose lipids in at P3 or P10 hADSCs overexpressing LYPLAL1-AS1 or control on day 10 after adipogenic induction. B. Cell proliferation of P3 hADSCs was tested by MTS when LYPLAL1-AS1 was knocked down. C. Cell proliferation of P3 and P10 hADSCs was tested by MTS when LYPLAL1-AS1 was overexpressed. D. Cell cycle analysis of hADSCs transfected with Lenti-NC or Lenti-LYPLAL1-AS1 at P10. (TIF 2176 KB)

**Additional file 6: Table S1**. Demographic data of healthy donors. **Table S2**. Primer sequences. **Table S3**. MIRLET7B promoter sequence (2000bp).

## Data Availability

The data that support the findings of this study are available from the corresponding author upon reasonable request.
