# Supplementary material for: LncRNA LYPLAL1-AS1 rejuvenates human adipose-derived mesenchymal stem cell senescence via transcriptional MIRLET7B inactivation
Source: Cell Biosci. 2022 Apr 21;12:45. doi: 10.1186/s13578-022-00782-x (PMC9022335; doi:10.1186/s13578-022-00782-x)
Supplement: Supplementary file 1 — Additional file 1. Methods and figure legends [file 13578_2022_782_MOESM1_ESM.doc]

**Supplementary material and method**

***Cell proliferation assay***

5 × 10^3^ cells were seeded into 96-well culture plates and incubated overnight; each group had five replicates. Cell proliferation was determined using the CellTiter 96 Aqueous One Solution Reagent Cell Proliferation Assay (MTS assay) (Promega, G3581). Briefly, the culture medium was removed, and 20 µl MTS solution was added to each well in 100μl culture medium. Cultures were incubated at 37°C for 1h under 95% humidity and 5% CO2. Optical density was measured at 490 nm using a microplate reader (Bio-Rad 680, Hercules, USA).

***Cell Cycle analysis***

Cell cycle analysis was performed with Cell Cycle and Apoptosis Analysis Kit (YEASEN, 40301ES50) followed by flow cytometry analysis. Briefly, hADSCs transfected with lenti-NC or lenti-LYPLAL1-AS1 at P10 were harvested, washed in PBS, and fixed at 4 °C with 70% ethanol for over 2h. Cells were washed with cold PBS and incubated at 37°C for 30 min with PI/RNase solution. Samples were analyzed with a flow cytometer (BD, San Jose, CA) equipped with a 488 nm laser, and cell cycles were analyzed by FlowJo_V10 software.

**Supplementary Figure Legends**

***Figure S1.*** ***Morphological, functional, and phenotype characteristics of hADSCs***

hADSC phenotypes at passage (A) 3 and (B) 10 detected by flow cytometry. Black curves represent isotype controls. hADSCs at passage 3 and 10 were induced for osteogenic differentiation and was detected by (C) ALP staining and (D) Alizarin red staining on day 5 and 12, respectively. hADSCs at passage 3 and 10 were cultured for adipogenic differentiation and was detected by (E) oil red O staining on day 10. scale bars: 200 µm.

***Figure S2***. Correlation analysis of LYPLAL1-AS1 expression level and age in healthy donors (n=42). data were analyzed using R software version 3.5.3.

***Figure S3***. Subcellular fractionation of LYPLAL1-AS1 in hADSCs followed by qRT-PCR. GAPDH and U6 mRNA served as cytoplasmic and nuclear control, respectively.

***Figure S4***. ***LYPLAL1-AS1 overexpression ameliorates cellular senescence, resulting in increased cell differentiation potential and increased cell proliferation.*** A. Oil red O staining of adipose lipids in at P3 or P10 hADSCs overexpressing LYPLAL1-AS1 or control on day 10 after adipogenic induction. B. Cell proliferation of P3 hADSCs was tested by MTS when LYPLAL1-AS1 was knocked down. C. Cell proliferation of P3 and P10 hADSCs was tested by MTS when LYPLAL1-AS1 was overexpressed. D. Cell cycle analysis of hADSCs transfected with Lenti-NC or Lenti-LYPLAL1-AS1 at P10.
