# Supplementary figures and images for: LncRNA LYPLAL1-AS1 rejuvenates human adipose-derived mesenchymal stem cell senescence via transcriptional MIRLET7B inactivation
Source: Cell Biosci. 2022 Apr 21;12:45. doi: 10.1186/s13578-022-00782-x (PMC9022335; doi:10.1186/s13578-022-00782-x)

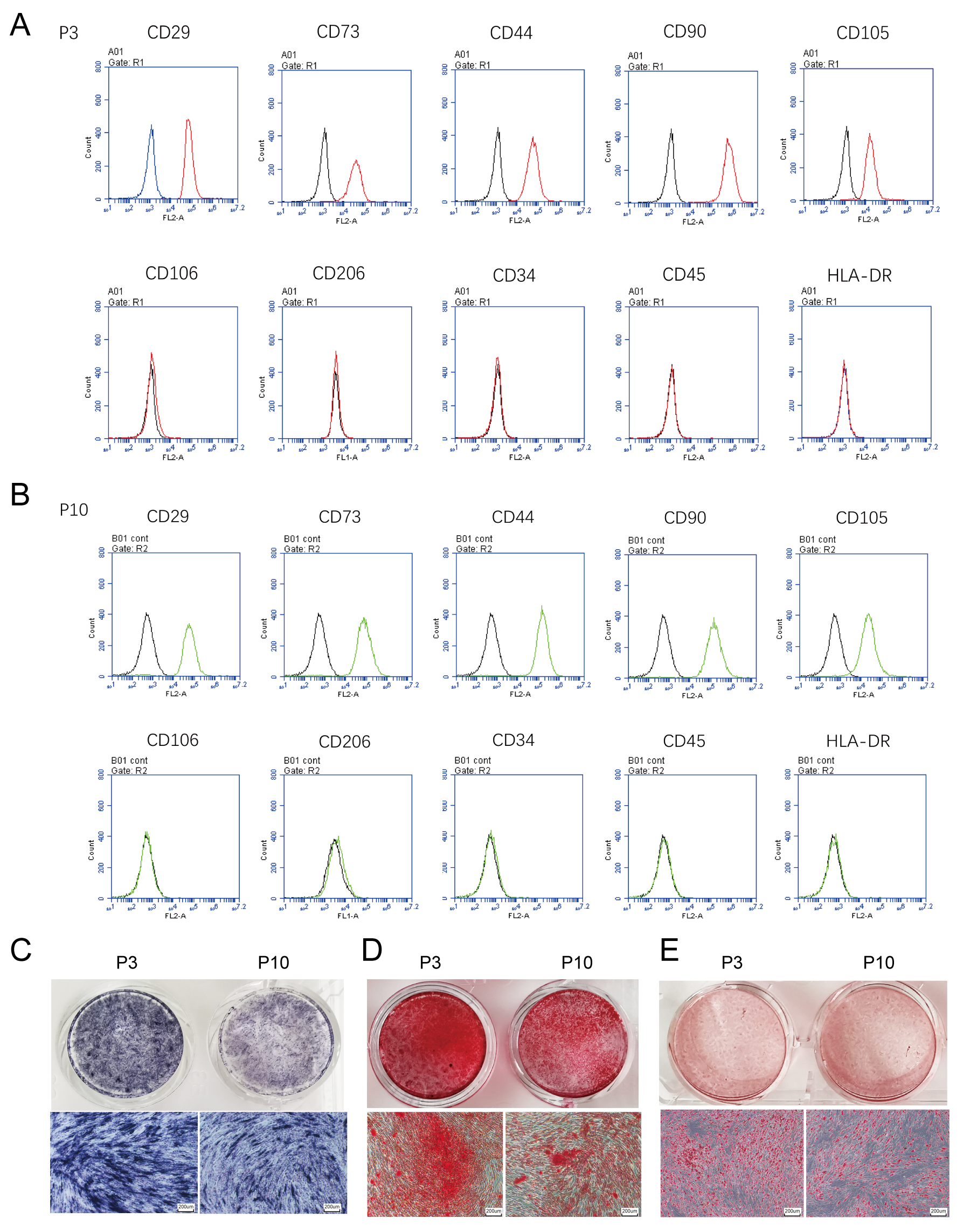

Supplement: Supplementary file 2 — Additional file 2: Figure S1. Morphological, functional, and phenotype characteristics of hADSCs [file 13578_2022_782_MOESM2_ESM.tif]

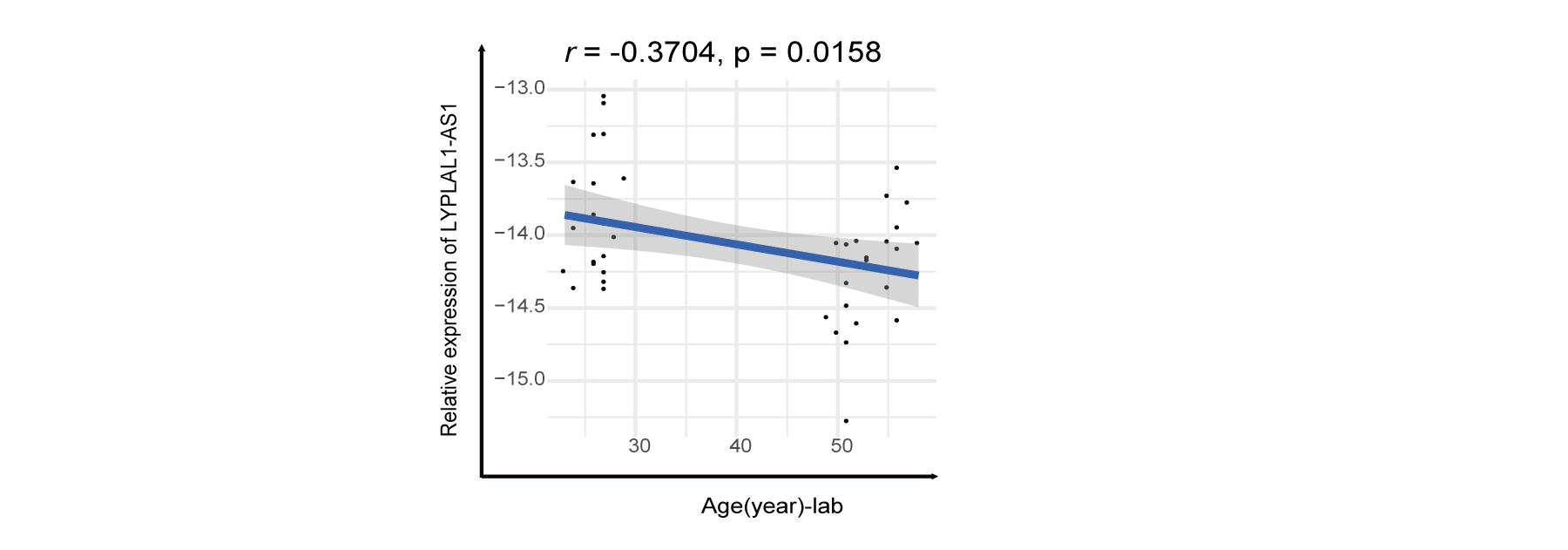

Supplement: Supplementary file 3 — Additional file 3: Figure S2. Correlation analysis of LYPLAL1-AS1 expression level and age in healthy donors (n=42). data were analyzed using R software version 3.5.3 [file 13578_2022_782_MOESM3_ESM.tif]

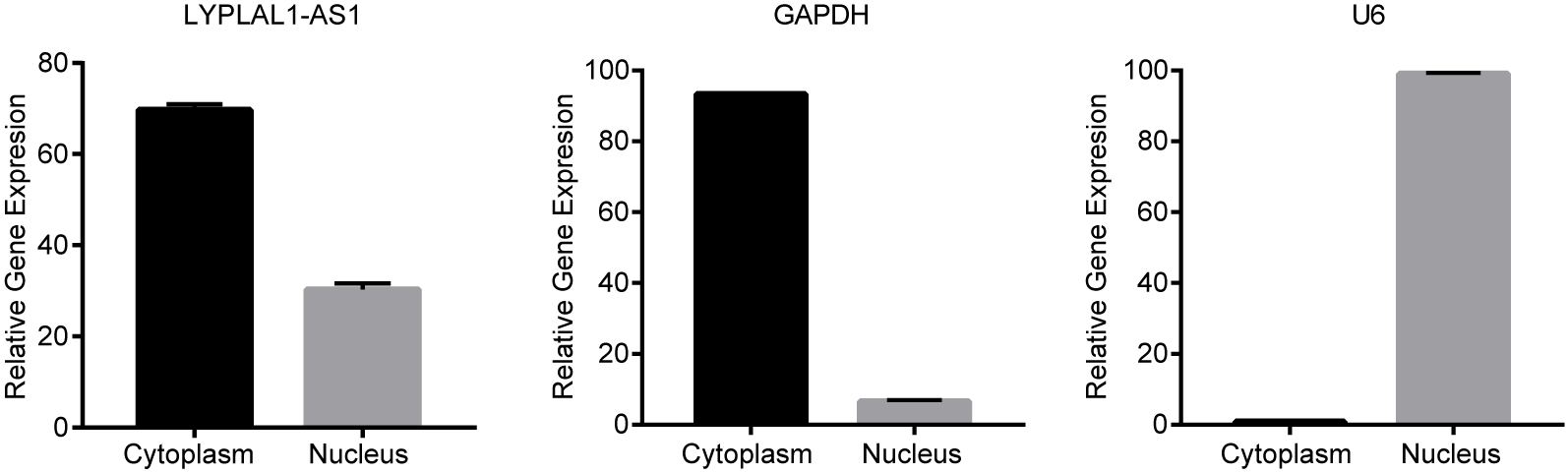

Supplement: Supplementary file 4 — Additional file 4: Figure S3. Subcellular fractionation of LYPLAL1-AS1 in hADSCs followed by qRT-PCR. GAPDH and U6 mRNA served as cytoplasmic and nuclear control, respectively. [file 13578_2022_782_MOESM4_ESM.tif]

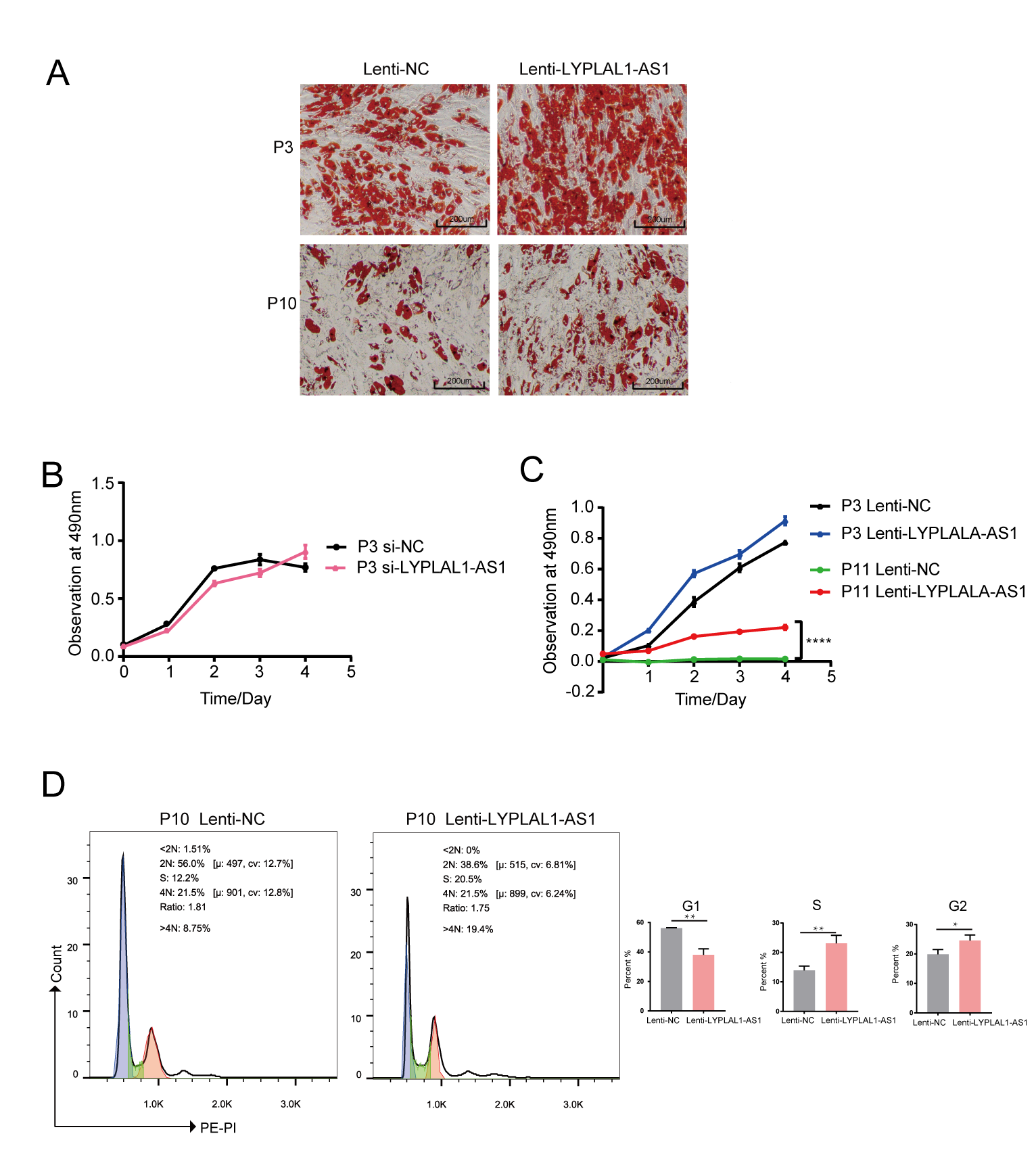

Supplement: Supplementary file 5 — Additional file 5: Figure S4. LYPLAL1-AS1 overexpression ameliorates cellular senescence, resulting in increased cell differentiation potential and increased cell proliferation. A. Oil red O staining of adipose lipids in at P3 or P10 hADSCs overexpressing LYPLAL1-AS1 or control on day 10 after adipogenic induction. B. Cell proliferation of P3 hADSCs was tested by MTS when LYPLAL1-AS1 was knocked down. C. Cell proliferation of P3 and P10 hADSCs was tested by MTS when LYPLAL1-AS1 was overexpressed. D. Cell cycle analysis of hADSCs transfected with Lenti-NC or Lenti-LYPLAL1-AS1 at P10. (TIF 2176 KB) [file 13578_2022_782_MOESM5_ESM.tif]
