# Supplementary material for: LncRNA LYPLAL1-AS1 rejuvenates human adipose-derived mesenchymal stem cell senescence via transcriptional MIRLET7B inactivation
Source: Cell Biosci. 2022 Apr 21;12:45. doi: 10.1186/s13578-022-00782-x (PMC9022335; doi:10.1186/s13578-022-00782-x)
Supplement: Supplementary file 6 — Additional file 6: Table S1. Demographic data of healthy donors. Table S2. Primer sequences. Table S3. MIRLET7B promoter sequence (2000bp). [file 13578_2022_782_MOESM6_ESM.doc]

**Supplementary Tables**

***Supplementary Table 1. Demographic data of healthy donors***

| **Subject** | **Young adult** | | **Middle age** | |
| --- | --- | --- | --- | --- |
|  | **Sex** | **Age (year)** | **Sex** | **Age (year)** |
| **1** | Female | 24 | Female | 49 |
| **2** | Male | 26 | Female | 50 |
| **3** | Female | 26 | Female | 50 |
| **4** | Male | 26 | Male | 51 |
| **5** | Female | 27 | Male | 51 |
| **6** | Female | 27 | Female | 52 |
| **7** | Female | 27 | Female | 52 |
| **8** | Female | 29 | Female | 55 |
| **9** | Female | 23 | Male | 58 |
| **10** | Female | 24 | Male | 51 |
| **11** | Female | 24 | Female | 51 |
| **12** | Female | 24 | Female | 51 |
| **13** | Female | 26 | Female | 53 |
| **14** | Male | 26 | Female | 53 |
| **15** | Male | 27 | Male | 55 |
| **16** | Male | 27 | Male | 55 |
| **17** | Female | 27 | Female | 56 |
| **18** | Female | 27 | Male | 56 |
| **19** | Female | 27 | Male | 56 |
| **20** | Female | 27 | Female | 56 |
| **21** | Female | 28 | Male | 57 |
| **Median age** | | 27 |  | 53 |
| **Mean age** | | 26 |  | 53 |

***Supplementary Table 2. Primer sequences.***

| **Gene** | **Primer sequence** (5’-3’) |
| --- | --- |
| LncRNA LYPLAL1-AS1 | F: GAGGAGGAGAAGCAAACTACAG |
|  | R: GACTCAGTCATGCCACTAAGG |
| P16 | F: GATCCAGGTGGGTAGAAGGTC |
|  | R: CCCCTGCAAACTTCGTCCT |
| P21 | F: CCCCTGCAAACTTCGTCCT |
|  | R: AAAGTCGAAGTTCCATCGCTC |
| LMNB1 | F: AAGCATGAAACGCGCTTGG |
|  | R: AGTTTGGCATGGTAAGTCTGC |
| miR-let-7b | F: GCGCGTGAGGTAGTAGGTTGT |
|  | R: AGTGCAGGGTCCGAGGTATT |
| U6 | F: AGTTTGGCATGGTAAGTCTGC |
|  | R: ACGCTTCACGAATTTGCGTGTC |
| GAPDH | F: GGTCACCAGGGCTGCTTTTA |
|  | R: GGATCTCGCTCCTGGAAGATG |
| miR-let-7b-RT | GTCGTATCCAGTGCAGGGTCCGAGGTATTCGCACTGGATACGACAACCAC |

**Supplementary Table 3. MIRLET7B promoter sequence (2000bp)**

| **sequence** (5’-3’) |
| --- |
| ATTTGGACCTCACTCTGCTGCCCCCTTGGCTGTGTGACATCCAGGTCACGTTGCCTCTCTGGGCCTCGGTCTCCTCACCTGTTTAAGAGGGGTTGACAGTCGTATCTGCCCCCTCAGCTTTTCCCCAGGAAGGTGGTAGCCACAATTAGCATTTGTTGAGGCTGACCCTGCACCAGGCCCAGGATAGGCGGGGCTTAGGGAGGCCCGTCTCTCGCCACGTTCCCCTGCTAGGGGAGCCCCGAGGCCCTCTCAGTGTCATCCTCATGCTACACTCTGTCCCAGCCCTGTGCGTCCCAAGCTAGGGCACTGAGTGTGCCAGCACCCGCAGGGACAGGCACTGGACCCTGGGTGGACCTGAGGGTCTGTGACTACCCCCCCAGCTGCTCTCCCCTAGAGGCCACTTCCCTCAAGGAAGGAAAGAACCTTCCCGCCACCTCCTGCAGTGCGGTCAGCTCAGGCCAGCCTGCACAGCAGGGCCAGAACCAGGGCCCCTGGGGAGGGATGCCTGCCTGCCCAGTGGGAGGAGACGGCACGCCCGTGAAGCCGCTACTCAGCCAGCCTGGGGGCCACGAGTGCTGCTTCTGGTGGCGCTGTGCGGGGAGGGAGGGGGCCGAGCAGGGTGGGCACTCGCATGCCTGTGTCTTGCTGGCCTTCGACAGATGACAGCCCTCCTCCTAGGGTCTCCAGTGCAGAGTTCCTTGGGGACATTATGGCCACTCCTGTCCAGATGAGAGGGAGCCGGCTGCCTGTGACAGCGTCGCAAAATGCCGCCAGGGCTTTCCCTCCCTCCTCCTTTCTCTCTTCCTCGTCCCTCTCTGGTTGGTGGTTTCCTGCAGGCTCCCGTCCCTGCTGGTGCTGGCCACAATGTCCCCACTCCCAGGGTTTCGGCGTCCCAGCCCCCTGCGCCCACCGCGCCTGCCCGCCAGAATCCCTGTGCCCTTGGTGCGTGTGGCCTGCCGAGCCTCGAGCCCCTGTTCTCCTCAGCCCTCTTTCCTCCCGCGTCCCCAGGAGGTGCCTCTGGAAGCCACGGAGTCCCATCGGCACCAAGACCGACTGCCCTTTGGGGTGAGGTAGTAGGTTGTATAGTTTGGGGCTCTGCCCTGCTATGGGATAACTATACAATCTACTGTCTTTCCTGAAGTGGCTGTAATATCTGCGGTGGACAGAGCGTCTGGAACCCTGGCTGGGAGCGGGCAGGGCCAGGTTTGGGGGCAGCCTTGGCAGCAGTCGGGGGCAGGGGCCGCCTACACTGAGAAGTCTGACAGGCCTAGGTGCCACTTGCTGTGTGACCTTGGACAGGCCCCTGATCTCTCTGGGTCTCAGTTTCCTCCTCTGTAAAATGGAGGCAAATGAGGATGGAAGGAGATGCAGTGTGGAGCATCGAGGGCAGAGGAGAGCTGAGCCGACCCCACCCTCTGCCCCAGCCGCACTGAGAGAGGCGATCCACGCAGCTGTTTGTCTGACCTCTGTCTCCCAACACTCCCCAACACTCCCCCCGCCATCAGGCCCAGGCTCATGGGTGGCCCTGAGCCGTACCCTCCACTGAGCACCAGGAGAAGGCACCGTGGGGCCAGGGGTGGCCGAGACGTTTGGAGGTCACAGGGCTGCGAGTATTGGCGTTGCCCATCACCCCAGGTTCCCAGCACGTGCCCCAGCCTGGCCAGCTCAGTGGCAGGGCCTCTGCCTGTGGAGGAAGGGAGGCCAAGGCACCTTTCCTGAGCAGGAAGTGAGAGGAACAGCTCTGCATACACTGGGTCCCACATGGCACAATCTGAAGGCAGACAGTGGCTCCTCTGTACCTGGGGAAACTGAGGCCCAGAGAGCCAGGGACTTCCCAAGACCAGCCAGCAGCAGCTGCCCCTTCCTGGGGTGCCATCTCCCCTGTCCCTCCTGCCCTGCGCCTGCCCAGCCCTCCTGCTCTGGTGACTGAGGACCGCCAGGCAGGGGCTGGTGCTGGGCGGGGGGCGGCGGGCCCTCCCGCAGTGCAAGGCCGGGCCTGG |
